# Rheumatoid arthritis and psoriatic arthritis: is the disease impact different? A large matching study at diagnosis and after 1 year of treatment

**DOI:** 10.1136/rmdopen-2024-005143

**Published:** 2025-03-12

**Authors:** Selinde V J Snoeck Henkemans, Anne-Fleur van den Biggelaar, Elise van Mulligen, Sytske Anne Bergstra, Jolanda J Luime, Marc R Kok, Ilja Tchetverikov, Maikel van Oosterhout, Jos H van der Kaap, Annette H M van der Helm-van Mil, Marijn Vis, Pascal H P de Jong

**Affiliations:** 1 Department of Rheumatology, Erasmus MC, Rotterdam, The Netherlands; 2 Department of Rheumatology, Leiden University Medical Center, Leiden, The Netherlands; 3 Department of Rheumatology and Clinical Immunology, Maasstad Hospital, Rotterdam, The Netherlands; 4 Department of Rheumatology, Albert Schweitzer Hospital, Dordrecht, The Netherlands; 5 Department of Rheumatology, Groene Hart Hospital, Gouda, The Netherlands; 6 Department of Rheumatology, Admiraal De Ruyter Hospital, Goes, The Netherlands

**Keywords:** Rheumatoid Arthritis, Psoriatic Arthritis, Patient Reported Outcome Measures

## Abstract

**Objective:**

Rheumatoid arthritis (RA) and psoriatic arthritis (PsA) are currently treated similarly. However, it is unclear which patient-reported outcome (PRO) domains need specific attention in the management of RA and PsA. Therefore, we aimed to determine the difference in disease impact between matched RA and PsA patients at diagnosis and after 1 year in two different regions.

**Methods:**

RA patients from the treatment in the Rotterdam Early Arthritis CoHort trial (tREACH), PsA patients from the Dutch southwest Early PsA cohoRt (DEPAR) and RA and PsA patients from the Leiden Early Arthritis Clinic (EAC) were included. The difference in disease impact between RA and PsA was measured with the following PROs: pain (Visual Analogue Scale (VAS), 0–100), fatigue (VAS), activity limitation (Health Assessment Questionnaire-Disability Index) and health impact (general health (VAS) and 36-item Short-Form Health Survey (SF-36)). Propensity scores were used to match RA and PsA patients, after which inverse probability weights (IPWs) were calculated. IPW-weighted linear regression models were used to measure PRO differences.

**Results:**

391 RA patients from tREACH, 416 PsA patients from DEPAR, 702 RA and 99 PsA patients from the EAC were included. At diagnosis, PsA-DEPAR patients scored 5.04 units worse (95% CI 2.21 to 7.87) on SF-36 mental health compared with RA-tREACH patients. This difference still existed after 1 year of treatment (3.88 (95% CI 1.90 to 5.86)). PsA-EAC patients had more activity limitations after 1 year of treatment compared with RA-EAC patients (−0.30 (95% CI −0.50 to −0.10)). No significant differences were present in the other PRO domains.

**Conclusion:**

The disease impact of early RA patients is similar to matched early PsA patients on most PRO domains, except for mental health and functional limitations, which were worse in PsA after 1 year of treatment.

**WHAT IS ALREADY KNOWN ON THIS TOPIC**

- Rheumatoid arthritis (RA) and psoriatic arthritis (PsA) both have a significant impact on patients’ lives. Currently, both diseases are treated in a similar manner, following the treat-to-target principle. However, it is unclear which patient-reported outcome domains need specific attention in the management of both diseases.

**WHAT THIS STUDY ADDS**

- When early RA and oligoarticular and polyarticular PsA patients are matched on demographics and disease activity, they have a similar disease impact with respect to pain, fatigue and functional ability at diagnosis. After 1 year of treatment, the disease impact is still similar with respect to pain and fatigue.
- In contrast, PsA patients have worse mental health compared with RA patients at diagnosis. After 1 year of treatment, mental health is still worse, but now PsA patients also have more functional impairment compared with RA patients.

**HOW THIS STUDY MIGHT AFFECT RESEARCH, PRACTICE OR POLICY**

- Healthcare providers should be aware of the greater mental health impact and functional impairment in PsA patients compared with RA patients.

## Introduction

 Rheumatoid arthritis (RA) and psoriatic arthritis (PsA) are both characterised by joint inflammation.^1^,^3^ Nevertheless, the clinical manifestations of RA and PsA differ significantly. For example, PsA patients often have a broad spectrum of manifestations in addition to arthritis, such as psoriasis, enthesitis, uveitis, sacroiliitis, dactylitis and inflammatory bowel disease.^2 3^ Despite improved clinical outcomes in RA and PsA, both diseases continue to significantly impact patients’ lives.^4^,^8^ Whether the disease impact differs between RA and PsA at presentation and after treatment is still unclear. Investigating the difference in impact might help healthcare providers become more aware of the specific domains that need attention in the management of both diseases. This is especially relevant because a dual treat-to-target management approach is currently advocated, in which healthcare providers should strive for (1) control of the disease, and thus inflammation and (2) control of the illness, that is, controlling the impact of the disease on patients’ lives.^9 10^ The disease impact can be measured with patient-reported outcomes (PROs). The International Consortium for Health Outcomes Measurement (ICHOM) has agreed on the most relevant PRO domains for patients with inflammatory arthritis. These domains are pain, fatigue, activity limitation, overall emotional and physical health impact, work/school/housework ability and productivity.^11^

Previous studies that compared the disease impact of RA and PsA patients have shown contradictory results. Sokoll and Helliwell, for example, showed that health-related quality of life (HRQoL) and functional impairment were similar between both diseases after matching for disease duration.^12^ This result was found despite the fact that PsA patients used less disease-modifying anti-rheumatic drugs (DMARDs) and had less radiographic damage.^12^ The aforementioned results were reconfirmed by Borman *et al*.^13^ On the other hand, Geijer *et al* found that after 5 years of follow-up, early RA patients had a HRQoL that was comparable to the general population, while the HRQoL of early PsA patients was still impaired.^14^ In addition, a cross-sectional study in established RA and PsA patients has shown that RA patients felt less fatigued and had less (joint) pain compared with PsA patients.^15^ The crucial drawback of the aforementioned studies is that the patients were not matched on demographics and disease activity, which could be of additional value in determining the difference in disease impact.

Therefore, we set out to determine the difference in disease impact between matched RA and PsA patients. Due to the broader spectrum of manifestations in PsA, we hypothesise that the disease impact of PsA is larger than RA. Accordingly, our aim is to investigate the difference in disease impact, measured with four domains of the ICHOM-recommended PROs, namely pain, fatigue, activity limitation and health impact, between matched early RA and PsA patients from two different regions in the Netherlands, at diagnosis and after 1 year of treatment.

## Methods

### Patients

For this cross-sectional study, data from the treatment in the Rotterdam Early Arthritis CoHort trial (tREACH), the Dutch southwest Early PsA cohoRt (DEPAR) and the Leiden Early Arthritis Clinic (EAC) were used.

The tREACH is a single-blinded, multicentre, randomised controlled trial. The study was conducted in eight rheumatology centres in the south-west of the Netherlands. Eligible patients had a symptom duration of <1 year and arthritis in ≥1 joints.^16^ Patients were included between July 2007 and June 2013. After inclusion, patients were randomised in different initial treatment strategies. Patients received either (I) methotrexate, including DMARD combination therapies with or without glucocorticoid bridging therapy; (II) hydroxychloroquine or (III) non-steroid anti-inflammatory drugs*/*glucocorticoids as initial treatment. Treatment was intensified every 3 months if the Disease Activity Score was ≥2.4. For this study, we selected all patients who fulfilled the 1987 and/or 2010 criteria for RA.^17 18^

The DEPAR started in August 2013 and is an ongoing, real-world, prospective cohort study, carried out in 14 hospitals in the south-western part of the Netherlands. Newly diagnosed, DMARD-naïve PsA patients, according to the treating rheumatologist, are included.^8^ Patients were treated according to (inter)national guidelines. For this study, data up to March 2023 were used from all consecutive PsA patients with oligoarthritis (2–5 joints) or polyarthritis (>5 joints) as their main clinical symptom according to the treating rheumatologist. Besides arthritis, patients could also have other PsA manifestations, that is, psoriasis, enthesitis, dactylitis and axial disease.

The EAC is a cohort that includes patients with arthritis in ≥1 joints and a symptom duration of ≤2 years in the Leiden area.^19^ Recruitment started in February 1993 and is ongoing. On inclusion, patients were treated according to (inter)national guidelines. For this study, all consecutive patients with an RA or PsA diagnosis who were included between 2010 (the time when the 36-item Short Form Health Survey (SF-36) was first administered in the EAC) and 2021 were selected. RA diagnosis was evaluated after 1-year follow-up and was defined according to the 1987/2010 criteria plus a clinical diagnosis by an experienced rheumatologist. PsA diagnosis was made by the treating rheumatologist.

Written informed consent was obtained, according to the Declaration of Helsinki, of all participants in the tREACH, DEPAR and EAC. Further details on the studies can be found elsewhere.^8 16 19^

### Data collection

In the tREACH and DEPAR, patients were assessed at baseline and every 3 months thereafter during the first year of follow-up. In the EAC, patients were assessed at baseline, after 4 months and yearly thereafter. At each visit, a physical examination was performed by research nurses to assess swollen joint count (SJC) and tender joint count (TJC). For PsA patients in DEPAR, the physical examination also included a skin assessment for psoriasis (body surface area, BSA), enthesitis assessment (Leeds Enthesitis Index, LEI) and a dactylitis count. In addition, blood samples were taken to measure C reactive protein (CRP) in both RA and PsA, and anti-citrullinated protein and rheumatoid factor (RF) in RA.

Medication data, including biological or targeted synthetic DMARD (b/tsDMARD) use, were collected from patients’ medical files in the tREACH and DEPAR. In the EAC, no data on b/tsDMARD usage were available.

### Outcomes

PROs measuring the following domains were collected: pain, fatigue, activity limitations and health impact. In the tREACH study, pain was measured with a Numeric Rating Scale (NRS), ranging from 0 to 10. The NRS was transformed to a 0–100 scale to make scores comparable between cohorts. Higher scores indicate more pain.^20^ In the EAC and DEPAR, pain was measured with a Visual Analogue Scale (VAS), ranging from 0 to 100 mm.^21^

Fatigue was measured with a 0–100 mm VAS in both the tREACH and EAC.^22^ In DEPAR, one question from the Bristol Rheumatoid Arthritis Fatigue Multi-Dimensional Questionnaire, that is, ‘What was your average level of fatigue during the past 7 days?’, which is an NRS ranging from 0 to 10, was used.^23^ Again, the NRS was transformed to a 0–100 scale to make the fatigue scores comparable. Higher scores represent more severe fatigue.^22^

For activity limitations, the Health Assessment Questionnaire-Disability Index (HAQ-DI) was used in all cohorts. HAQ-DI scores range from 0 to 3. Higher scores indicate more functional impairment.^24^

Health impact was measured with a VAS general health and the SF-36 in all three cohorts. Higher VAS scores indicate a poorer health status.^21^ The SF-36 measures eight domains, that is, bodily pain, vitality, mental health, general health, social functioning, physical functioning, role emotional and role functional. Each domain is scored on a 0–100 scale, and higher scores indicate a better HRQoL. A Physical Component Score (PCS) and Mental Component Score (MCS) were calculated by standardising the domain scores based on Dutch population norms.^25 26^

### Statistical analysis

To compare RA and PsA patients on PROs at baseline and after 1 year, corrections were made for differences in baseline covariates. This was done by using inverse probability weighting (IPW) based on propensity scores.^27^ In this study, the propensity score (range 0–1) can be seen as the patient’s probability of being in the RA or PsA group, conditional on observed baseline covariates.^28^

Variable selection for the propensity score was based on prior knowledge of variables related to the outcome, availability of the variables in all three datasets and missingness (variables with ≥15% missingness were excluded to maintain a larger number of patients).^29^ Consequently, at baseline, 93%, 73%, 82% and 72% of the total number of available patients in the tREACH, DEPAR, EAC (RA) and EAC (PsA) were included in our study, respectively. Of the included patients, the number of missing data per PRO is shown in online supplemental table S1. At baseline, the number of missing data was low in all cohorts. After 1 year, the number of patients with missing data had increased.

For the analysis at baseline, we selected the following variables: age, sex, symptom duration, SJC-44/66, TJC-53/68, smoking behaviour (yes/no), CRP and education level. For the analysis after 1 year, propensity scores were calculated using the same baseline variables, combined with baseline values of the PROs. For the comparison of RA and PsA patients within the EAC, the same variables were used, except for education level because of ≥15% missingness. Since distal interphalangeal joint involvement is common in PsA, the choice was made to use the SJC-66 and TJC-68 in PsA and to compare these with the SJC-44 and TJC-53 in RA.^30^ Propensity scores were estimated using logistic regression models and were calculated separately for the comparison of RA-tREACH and PsA-DEPAR, and RA-EAC and PsA-EAC.

In order to achieve balance between baseline covariates of the cohorts, multiple different specifications of the propensity scores were performed, which included categorising and the addition of higher order terms of variables (SJC, TJC and CRP).^31^ Online supplemental table S2 shows in which form SJC, TJC and CRP were used in the final specifications of the propensity scores. We managed to achieve common support for all but one propensity score, indicating that each patient has a positive probability of being in the RA or PsA group. For the comparison between RA-EAC and PsA-EAC after 1 year of treatment, no common support was achieved. Therefore, eight PsA-EAC patients who were in the non-overlapping region were disregarded from further analyses.

Thereafter, IPW was applied, which ensured that all available data were used.^32^ We set the RA patients as 1, and PsA patients as p/(1-p), with p as the probability that a patient is in the RA group.^32^ The IPWs were used as weighting factors in linear regression models to determine the difference in disease impact between RA and PsA. In the linear regression models, the type of disease (RA or PsA) was set as the independent variable and the aforementioned PROs were set as dependent variables. Robust SEs were used to overcome mis-specification of SEs in the weighted regression models.^33^

To assess the comparability of the RA and PsA patients after IPW, three matching statistics were used. The first is Rubin’s B, which represents the standardised difference of means between the RA and PsA groups. The second statistic is Rubin’s R, which represents the ratio of the variances of the propensity scores in the RA and PsA patients. Preferably, Rubin’s B should be <25% and Rubin’s R between 0.5 and 2.0. The last statistic is the standardised bias, calculated for each covariate separately, which is the difference in sample means in the RA and PsA groups divided by the square root of the average of the sample variances.^27 34^ For covariates where the standardised bias was >10% after IPW, indicating that groups were not fully balanced, a double robust IPW was performed, meaning that these covariates were added to the weighted regression models for an additional correction.^35^

Linear regression models with diagnosis as independent variable and PROs as dependent variable were repeated for the crude data, which is comparable to clinical practice.

The size of the betas in the regression models was compared with the minimal clinically important difference (MCID) to determine if the differences in PROs between RA and PsA were clinically relevant.^36 37^ For example, the MCID for the HAQ-DI is 0.22, while the MCID for the SF-36 MCS and PCS is 2.5–5.

Finally, a sensitivity analysis was performed in which propensity scores were trimmed to 0.1–0.9. This reduces bias by removing extreme propensity scores. After trimming, the propensity scores were re-estimated to improve covariate balance.^38 39^ In a second sensitivity analysis, we show crude estimates of the PROs at baseline and after 1 year of treatment in RA and PsA patients.

All analyses were performed in Stata V.18.0. To adjust for multiple testing, a Bonferroni correction was applied by multiplying the p values with the 48 performed tests. A p≤0.05 was considered statistically significant.

## Results

A total of 1093 RA (tREACH n=391; EAC n=702) and 515 PsA patients (DEPAR n=416; EAC n=99) were included. RA patients were more often women (67% and 64% in RA vs 46% and 39% in PsA). PsA patients in DEPAR had a longer symptom duration (median (IQR) 8.3 (3.6–28.3) months) compared with the other cohorts. SJC and TJCs were higher in RA compared with PsA (table 1). After IPW, baseline characteristics of RA and PsA patients were comparable (tables2^3^). Baseline characteristics and PROs, which were used for calculating the IPW at 1 year, are shown in online supplemental table S3–S4.

**Table 1:** Baseline characteristics of RA and PsA patients

	RA—tREACH (n=391)	PsA—DEPAR (n=416)	RA—EAC (n=702)	PsA—EAC (n=99)
Demographic characteristics				
Age (years), mean (SD)	53.4 (14.2)	51.6 (14.0)	59.1 (14.4)	49.9 (15.4)
Sex (female), n (%)	260 (67)	190 (46)	450 (64)	39 (39)
Symptom duration (months), median (IQR)	4.7 (2.9–6.9)	8.3 (3.6–28.3)	3.1 (1.6–7.0)	3.6 (1.5–8.8)
Current smoker, n (%)	114 (29.2)	95 (22.8)	152 (21.7)	17 (17.2)
Education level*, n (%)				
Low	224 (57.3)	171 (41.1)	334 (50.8)	33 (36.7)
Intermediate	105 (26.9)	141 (33.9)	177 (26.9)	32 (35.6)
High	62 (15.9)	104 (25.0)	146 (22.2)	25 (27.8)
Disease activity				
SJC-44/66, median (IQR)	7.0 (4.0–12.0)	3.0 (2.0–6.0)	5.0 (2.0–10.0)	3.0 (1.0–5.0)
TJC-53/68, median (IQR)	10.0 (5.0–15.0)	5.0 (2.0–9.0)	8.0 (4.0–13.0)	5.0 (2.0–8.0)
CRP, median (IQR)	7.0 (3.5–20.0)	5.1 (1.0–14.0)	9.0 (3.3–23.0)	4.4 (3.0–13.5)
Psoriasis, n (%)		303 (84.9)		76 (76.8)
BSA (%) in case of psoriasis, median (IQR)		3 (1–5)		
Enthesitis, n (%)		149 (35.8)		
LEI in case of enthesitis, median (IQR)		2 (1–3)		
Dactylitis, n (%)		75 (18.1)		
Chronic back pain <45 years, n (%)		133 (33)		
ACPA positivity, n (%)	197 (50.4)		332 (47.3)	
RF positivity, n (%)	195 (49.9)		386 (55.0)	

* Education level was defined according to the Organisation for Economic Co-operation and Development: low, below upper secondary level; intermediate, upper secondary level; high, tertiary education.

ACPA, anticitrullinated protein antibody; BSA, body surface area; CRP, C reactive protein; DEPAR, Dutch southwest Early PsA cohoRt; EAC, Leiden Early Arthritis Clinic; LEI, Leeds Enthesitis Index; PsA, psoriatic arthritis; RA, rheumatoid arthritis; RF, rheumatoid factor; SJC-44/66, 44/66 swollen joint count; TJC-53/68, 53/68 tender joint count; tREACH, treatment in the Rotterdam Early Arthritis CoHort trial.

**Table 2:** Baseline characteristics before and after IPW for RA (tREACH) and PsA (DEPAR) patients

	Before IPW			After IPW	
	RA (n=391)	PsA (n=416)	SB (%)	PsA (n=416)	SB (%)
Demographic characteristics					
Age (years)	53.4	51.3	14.7	54.1	−4.8
Sex, female (%)	67.3	47.5	40.8	67.4	−0.1
Symptom duration (months)	5.3	31.2	−61.3	4.7	1.6
Current smoker (%)	29.8	22.1	17.5	32.8	−7.0
Education level* (%)					
Low	56.9	40.0	34.8	60.4	−7.0
Intermediate	27.5	33.3	−12.6	23.4	8.8
High	15.6	27.0	−28.0	16.2	−1.5
Disease activity					
SJC-44/66	8.7	4.7	72.0	9.3	**−11.6**
TJC-53/68					
TJC-53/68 ≤1 (%)	7.8	18.0	−30.6	5.3	7.5
TJC-53/68 2–4 (%)	15.9	31.3	−36.9	14.8	2.7
TJC-53/68 ≥5 (%)	76.3	50.7	55.1	79.9	−7.7
CRP					
CRP ≤10	59.1	66.9	−16.2	60.3	−2.4
CRP >10	40.9	33.1	16.2	39.7	2.4

Matching statistics	Before IPW	After IPW
Mean bias	33.6	5.0
Rubin’s B	77.3	25.7
Rubin’s R	0.02	0.66

Results shown are means or as stated otherwise.

In order to achieve better matching between RA and PsA, TJC was categorised into ≤1, 2–4 or ≥5 tender joints, and CRP was dichotomised into ≤10 and >10. For RA, the SJC with 44 joints and TJC with 53 joints were used, whereas for PsA the SJC with 66 joints and TJC with 68 joints were used.

SB is the absolute standardised bias, also called absolute standardised mean difference (in %). Rubin’s B is the absolute standardised difference of means between the RA and PsA groups. Rubin’s R is the ratio of the group variances of the propensity score of the two groups. When optimal balance is achieved, SB is below 10%, Rubin’s B is below 25, and Rubin’s R is between 0.5 and 2. Since optimal balance was not achieved, in the double robust method, an extra correction was done on top of the weighting for the variable with an SB above 10% after weighting. This variable was SJC and is indicated in bold.

* Education level was defined according to the Organisation for Economic Co-operation and Development: low, below upper secondary level; intermediate, upper secondary level; high, tertiary education.

CRP, C reactive protein; DEPAR, Dutch southwest Early PsA cohoRt; IPW, inverse probability weighting; PsA, psoriatic arthritis; RA, rheumatoid arthritis; SB, standardised bias; SJC-44/66, 44/66 swollen joint count; TJC-53/68, 53/68 tender joint count; tREACH, treatment in the Rotterdam Early Arthritis CoHort trial.

**Table 3:** Baseline characteristics before and after IPW for RA (EAC) and PsA (EAC) patients

	Before IPW			After IPW	
	RA (n=702)	PsA (n=99)	SB (%)	PsA (n=99)	SB (%)
Demographic characteristics					
Age (years)	58.9	49.4	64.5	63.7	**−32.1**
Sex, female (%)	63.4	40.1	47.7	75.2	**−24.1**
Symptom duration (months)	7.0	9.8	−16.1	5.4	9.6
Current smoker (%)	21.4	18.5	7.1	28.5	**−17.6**
Disease activity					
SJC-44/66					
SJC-44/66 ≤1 (%)	14.4	25.4	−27.6	13.0	3.6
SJC-44/66 2–4 (%)	29.9	45.4	−32.2	29.8	0.3
SJC-44/66 ≥5 (%)	55.6	29.2	55.3	57.2	−3.3
TJC-53/68					
TJC-53/68 ≤1 (%)	8.2	15.4	−22.3	7.0	3.9
TJC-53/68 2–4 (%)	21.0	29.2	−18.9	16.2	**11.1**
TJC-53/68 ≥5 (%)	70.7	55.4	32.1	76.8	**−12.7**
CRP					
CRP ≤10	53.4	64.9	−23.6	53.8	−1.0
CRP >10	46.6	35.1	23.6	46.2	1.0

Matching statistics	Before IPW	After IPW
Mean bias	30.9	10.0
Rubin’s B	96.5	46.8
Rubin’s R	0.81	0.70

Results shown are means or as stated otherwise.

In order to achieve better matching between RA and PsA, SJC and TJC were categorised into ≤1, 2–4 or ≥5 swollen/tender joints and CRP was dichotomised into ≤10 and >10. For RA, the SJC with 44 joints and TJC with 53 joints were used, whereas, for PsA, the SJC with 66 joints and TJC with 68 joints were used.

SB is the absolute standardised bias, also called absolute standardised mean difference (in %). Rubin’s B is the absolute standardised difference of means between the RA and PsA group. Rubin’s R is the ratio of the group variances of the propensity score of the two groups. When optimal balance is achieved, SB is below 10%, Rubin’s B is below 25, and Rubin’s R is between 0.5 and 2. Since optimal balance was not achieved, in the double robust method, an extra correction was done on top of the weighting for the variables with an SB above 10% after weighting. These variables were age, sex, smoking status and TJC and are indicated in bold.

CRP, C reactive protein; EAC, Leiden Early Arthritis Clinic; IPW, inverse probability weighting; PsA, psoriatic arthritis; RA, rheumatoid arthritis; SB, standardised bias; SJC-44/66, 44/66 swollen joint count; TJC-53/68, 53/68 tender joint count.

### Pain (VAS, 0–100 mm)

**Figure 1:**
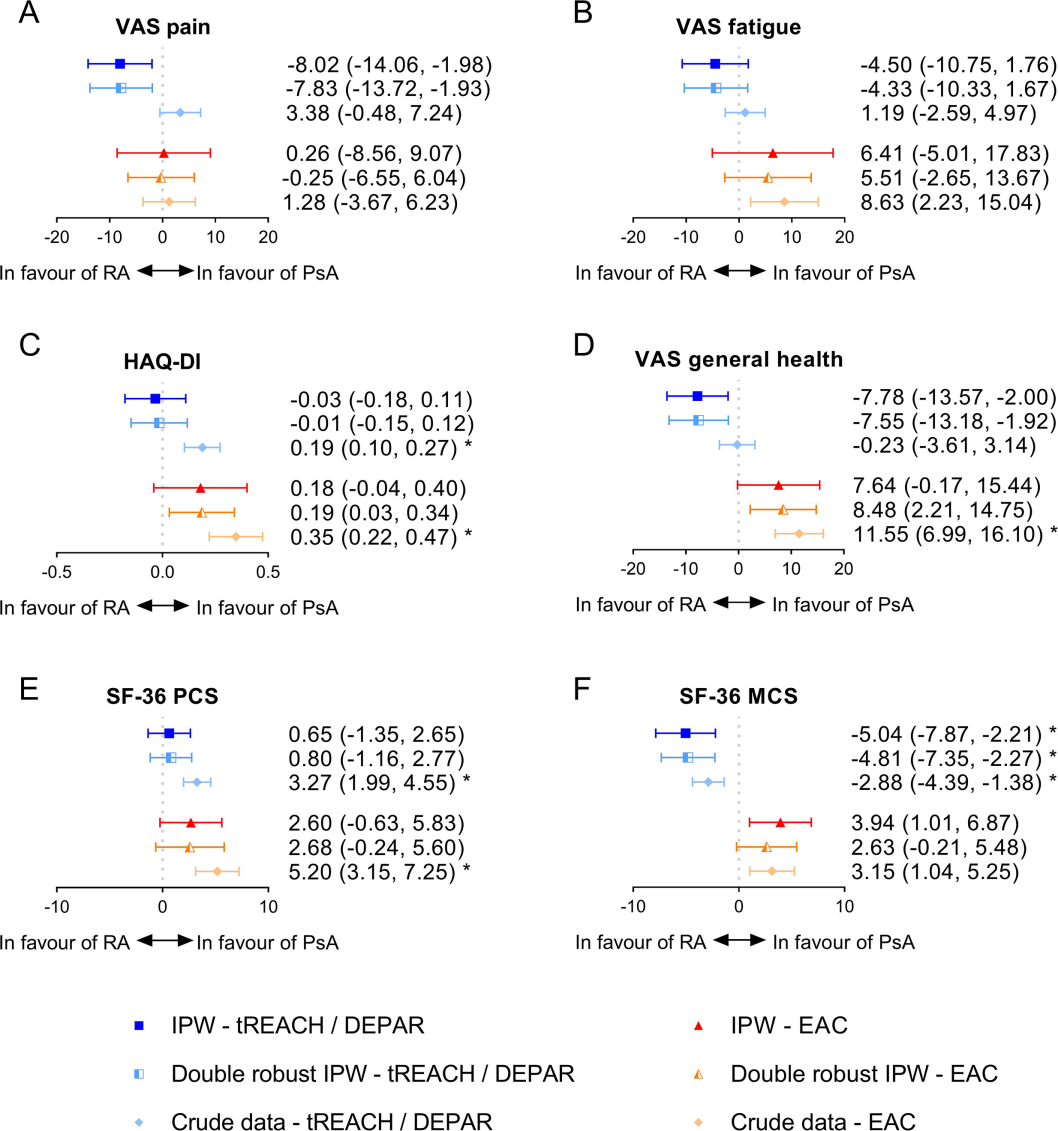
Differences in patient-reported outcomes between RA and PsA patients at diagnosis. Values shown are the mean differences in PROs between RA and PsA at diagnosis with the corresponding 95% CI. For example, RA-tREACH patients scored on average 8.02 points lower on a VAS pain compared with PsA-DEPAR patients at baseline. For readability purposes, negative differences in SF-36 PCS and MCS values are shown as positive differences and vice versa, so that a negative difference is in favour of RA and a positive difference in favour of PsA. Since optimal balance was not achieved in the RA-tREACH versus PsA-DEPAR, in the double robust method, an extra correction was done on top of the weighting for swollen joint count. In the comparison of the RA-EAC versus PsA-EAC, optimal balance was also not achieved, and additional corrections were done for age, sex, smoking status and tender joint count. *A significant difference (p≤0.05) after Bonferroni correction. DEPAR, Dutch southwest Early PsA cohoRt; EAC: Leiden Early Arthritis Clinic; HAQ-DI, Health Assessment Questionnaire–Disability Index; IPW, inverse probability weighting; PsA, psoriatic arthritis; RA, rheumatoid arthritis; SF36-MCS, 36-item Short Form Health Survey Mental Component Score; SF-36 PCS, SF-36 Physical Component Score; tREACH: treatment in the Rotterdam Early Arthritis CoHort trial; VAS, Visual Analogue Scale.

**Figure 2:**
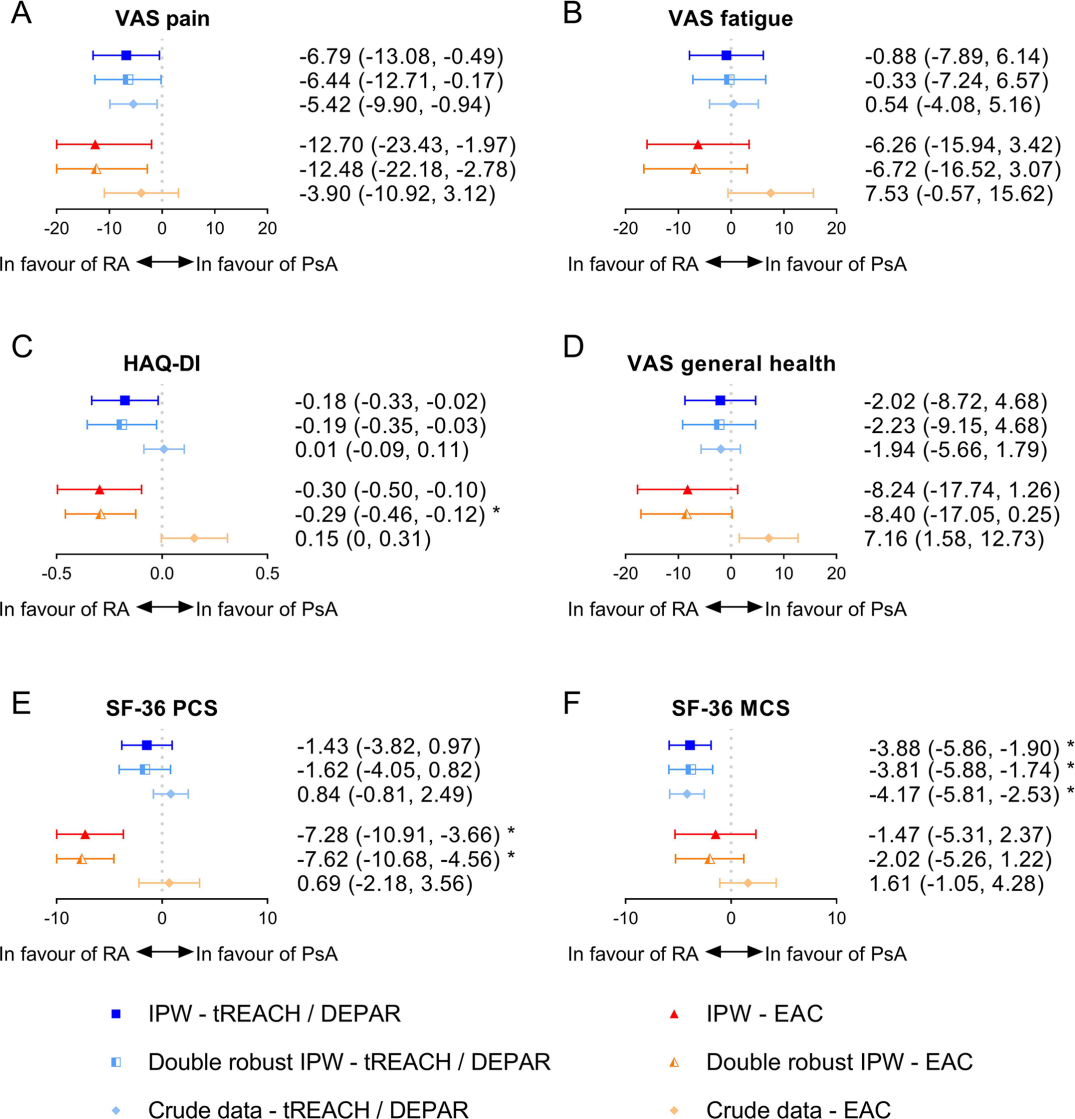
Differences in patient-reported outcomes between RA and PsA patients after 1 year of treatment. Values shown are the mean differences in PROs between RA and PsA after 1 year with the corresponding 95% CI. For example, RA-tREACH patients scored on average 6.79 points lower on a VAS pain compared with PsA-DEPAR patients after 1 year. Since optimal balance was not achieved in the RA-tREACH versus PsA-DEPAR, in the double robust method, an extra correction was done on top of the weighting for baseline CRP. In the comparison between the RA-EAC and PsA-EAC, optimal balance was also not achieved, and additional corrections were made for age, sex, baseline smoking status, baseline swollen joint count and baseline pain, fatigue and general health. *A significant difference (p≤0.05) after Bonferroni correction. CRP, C reactive protein; DEPAR, Dutch southwest Early PsA cohoRt; EAC: Leiden Early Arthritis Clinic; HAQ-DI, Health Assessment Questionnaire–Disability Index; IPW, inverse probability weighting; PsA, psoriatic arthritis; RA, rheumatoid arthritis; SF36-MCS, 36-item Short Form Health Survey Mental Component Score; SF-36 PCS, SF-36 Physical Component Score; tREACH: treatment in the Rotterdam Early Arthritis CoHort trial; VAS, Visual Analogue Scale.

RA-tREACH patients experienced less pain at diagnosis (−8.02 (95% CI −14.06 to −1.98)) compared with PsA-DEPAR patients after IPW, but the difference was not significant after Bonferroni correction (figure 1A). In the crude data, no differences in pain were seen between RA-tREACH and PsA-DEPAR patients. RA and PsA patients from the EAC experienced equal amounts of pain at diagnosis, both after IPW and in the crude data. After 1 year of treatment, RA-tREACH patients experienced less pain than PsA-DEPAR patients after IPW (−6.79 (95% CI −13.08 to −0.49)) and in the crude data, and similar results were found in the EAC (−12.70 (95% CI −23.43 to −1.97)) after IPW, but not in the crude data (figure 2A). The double robust IPW analyses showed similar results as the IPW analyses (figures1A  2A).

### Fatigue (VAS, 0–100 mm)

At diagnosis and after 1 year of treatment, no significant differences, after Bonferroni correction, were found in fatigue in both regions after IPW and in the crude data (figures1B  2B). The double robust IPW analyses showed similar results (figures1B  2B).

### Activity limitations (HAQ-DI, 0–3)

At diagnosis, functional impairments were similar between RA and PsA patients after IPW, while in the crude data of both regions, PsA patients had less functional impairments compared with RA patients (figure 1C). However, after 1 year, RA patients reported less functional impairments compared with PsA patients in the (double robust) IPW (figure 2C). RA-tREACH patients scored 0.18 units lower (95% CI −0.33 to −0.02) than PsA-DEPAR patients, while RA-EAC patients scored 0.30 units lower (95% CI −0.50 to −0.10) than PsA-EAC patients on the HAQ-DI. Only the results from the double robust analysis in the EAC were significant, while the MCID (>0.22) was exceeded for both the non-double robust and double robust analyses within the EAC.^36^ After 1 year, the crude data did not show a difference in functioning between RA and PsA patients in both regions (figure 2C).

### Health impact (general health, VAS 0–100 mm and SF-36 PCS/MCS, ~5–80)

Health impact was measured with a generic measure (general health) and a more specific measure (SF-36).

At diagnosis, general health was better in RA-tREACH patients compared with PsA-DEPAR (−7.78 (95% CI −13.57 to −2.00)) after IPW. However, general health was similar (non-double robust IPW) or worse (double robust IPW) in RA-EAC patients compared with PsA-EAC patients (7.64 (95% CI −0.17 to 15.44)) (figure 1D). In the crude data, general health was similar between RA-tREACH and PsA-DEPAR patients, and significantly worse in RA-EAC patients compared with PsA-EAC patients at diagnosis. After 1 year of treatment, the differences diminished for both the tREACH versus DEPAR (−2.02 (95% CI −8.72 to 4.68)) and the EAC versus EAC (−8.24 (95% CI −17.74 to 1.26)) after IPW (figure 2D). The double robust IPW analyses and crude data showed similar results, except for a (non-significant) better general health in the crude data of PsA-EAC patients (figures1D  2D).

With respect to the SF-36, no differences were seen on the SF-36 PCS between RA and PsA patients at diagnosis in both regions after (double robust) IPW, but RA patients had significantly worse SF-36 PCS scores in the crude data (figure 1E). After 1 year of treatment, no differences were found on the SF-36 PCS between RA-tREACH and PsA-DEPAR patients after IPW and in the crude data, but RA-EAC patients scored significantly better on the SF-36 PCS than PsA-EAC patients (7.28 (95% CI 3.66 to 10.91) after IPW (figure 2E). This difference also exceeded the MCID (≥2.5–5).^37^ In the crude data of the EAC, however, these differences were not observed (figure 2E).

For the SF-36 MCS, RA-tREACH patients scored significantly better than PsA-DEPAR patients at diagnosis after IPW (5.04 (95% CI 2.21 to 7.87)) and in the crude data (figure 1F). After 1 year of treatment, similar results were found for the comparison of the tREACH with DEPAR (3.88 (95% CI 1.90 to 5.86)) (figure 2F). The MCID (≥2.5–5), however, was only exceeded at diagnosis.^37^ In contrast, RA-EAC patients scored worse on the SF-36 MCS at diagnosis after IPW (−3.94 (95% CI −6.87 to −1.01)) and in the crude data (not significant after Bonferroni), but similar in the double robust IPW. No differences were seen on the SF-36 MCS in the EAC after 1 year (figures1F  2F).

### Treatment

In the first year, a similar number of patients were started on b/tsDMARDs in the tREACH and DEPAR, that is, 24% vs 22% after 6 months and 30% vs 31% after 1 year for RA-tREACH and PsA-DEPAR patients, respectively. In both RA-tREACH and PsA-DEPAR patients, PROs were worse after 1 year in those treated with b/tsDMARDs during the first year compared with those not treated with b/tsDMARDs, except for the SF-36 MCS (online supplemental table S5). Additional adjustment for b/tsDMARD use in the double robust IPW of the tREACH and DEPAR after 1 year did not change the results (results not shown).

### Sensitivity analysis

After trimming and re-estimating the propensity scores at baseline and after 1 year, the matching statistics showed slightly less bias for the comparison of RA-tREACH with PsA-DEPAR, but more bias for the comparison of RA-EAC with PsA-EAC. In general, both at diagnosis and after 1 year, PRO estimates showed similar results after trimming (online supplemental table S6–S9, figures S1,S2). However, the differences between RA and PsA patients on the SF-36 PCS and SF-36 MCS were no longer significant after Bonferroni correction (online supplemental figures S1,S2).

Crude estimates of the PROs at baseline and after 1 year before matching can be found in online supplemental table S10,S11. In these unmatched data, RA-tREACH patients scored numerically worse than PsA-DEPAR patients on pain, HAQ-DI and the SF-36 PCS at baseline, while they scored better on the SF-36 MCS. RA-EAC patients, on the other hand, scored worse on all PROs compared with PsA-EAC patients (online supplemental table S10). After 1 year of treatment, RA-tREACH and PsA-DEPAR patients scored similarly on most PRO domains. However, RA-EAC patients scored worse than PsA-EAC patients on fatigue, HAQ-DI, and general health, but better on pain after 1 year (online supplemental table S11).

We tried to identify whether the differences in SF-36 MCS between RA-tREACH and PsA-DEPAR patients at baseline and after 1 year could be due to the broader spectrum of manifestations in PsA. We, therefore, compared differences in the SF-36 MCS between PsA patients with >3% of their BSA affected by psoriasis with patients with ≤3% affected BSA, and between patients with and without enthesitis. We found that PsA-DEPAR patients with >3% BSA affected by psoriasis at baseline had worse mental HRQoL (median SF-36 MCS 48.6 (IQR 40–56)) at baseline compared with patients with ≤3% of their BSA affected (median 51.5 (IQR 41–57)). PsA-DEPAR patients with higher residual skin disease activity after 1 year (>3% BSA affected) also scored worse on mental HRQoL after 1 year compared with patients with ≤3% BSA affected (online supplemental table S12). With respect to enthesitis, we found that patients who had enthesitis (LEI>0) at baseline had worse mental HRQoL (median SF-36 MCS 46.4 (IQR 38–55)) compared with patients without enthesitis (median 51.5 (IQR 43–57), respectively) at baseline. Patients who had enthesitis after 1 year also scored worse on mental HRQoL after 1 year compared with patients without enthesitis (online supplemental table S12).

## Discussion

This study investigated whether the impact of the disease on patients’ lives differed at diagnosis and after 1 year of treatment between matched early RA and PsA patients from two different regions. At diagnosis PsA patients scored worse on pain, general health and the SF-36 MCS, but these differences were only significant for the SF-36 MCS (RA-tREACH vs PsA-DEPAR). The difference in the SF-36 MCS also exceeded the MCID, indicating worse mental HRQoL at diagnosis for PsA patients.^37^ After 1 year, the differences in disease impact between RA and PsA became smaller. A significant difference was again found for the SF-36 MCS (RA-tREACH vs PsA-DEPAR), which indicates that PsA patients still had more mental complaints even after 1 year of treatment.^37^ Interestingly, after 1 year of treatment, PsA patients had more functional limitations compared with RA patients (RA-EAC vs PsA-EAC).

Limited research on the difference in disease impact between RA and PsA has been done, with conflicting results. Some studies have found a higher disease impact in PsA than RA, in terms of pain, fatigue, general health and mental HRQoL, while others have described a higher impact in RA than PsA, with respect to functional impairment and physical HRQoL.^1540^,^43^ Interestingly, there are also studies showing comparable functional impairment and HRQoL between RA and PsA.^12 13^ While the aforementioned studies all compared established RA and PsA, some research has also been performed in early RA and PsA. One study found that early RA patients have a better physical HRQoL but a worse mental HRQoL at baseline compared with early PsA patients.^14^ Another study found that RA patients have both a worse physical and mental HRQoL at baseline.^44^ In contrast to the aforementioned studies, our study investigated the difference in disease impact between early RA and PsA patients who were matched on demographic characteristics and disease activity. Therefore, the aforementioned results from the literature are not easily comparable to our study.

In our study, mental HRQoL was worse in PsA-DEPAR patients compared with RA-tREACH patients at diagnosis and after 1 year. We cannot be sure what caused this difference, but skin involvement in PsA could be one of the causes. Psoriasis can negatively influence a patient’s body image and self-confidence, which could lead to mental complaints.^45^ Previous research has shown that the severity of psoriatic lesions is associated with poor mental health.^43^ At baseline, psoriasis was present in 85% of PsA-DEPAR patients, and patients with >3% of their BSA affected scored worse on the SF-36 MCS than patients with less severe psoriasis, which might explain the observed differences. In addition, the presence of enthesitis could be of influence, as patients with enthesitis scored worse on the SF-36 MCS. However, no significant differences in mental health were observed in the EAC between RA and PsA patients. We have no information on the severity of the psoriasis, nor on the presence of enthesitis in the EAC, and we, therefore, do not know whether psoriasis severity and the presence of enthesitis differ between DEPAR and the EAC and whether this could explain the different results in the two regions.

After 1 year of treatment, PsA-EAC patients had more functional impairment and worse physical HRQoL than RA-EAC patients. The cause of this difference remains unclear. Possible explanations include differences in treatment and comorbidities between RA and PsA patients and involvement of entheses and the spine in PsA. Unfortunately, no data were available on these topics in the EAC. To summarise, the presence of extra-articular manifestations in PsA-DEPAR could be the reason for the differences in disease impact between RA-tREACH and PsA-DEPAR patients at diagnosis and after treatment, but the cause of the differences found between RA-EAC and PsA-EAC remains unclear.

As expected, the crude data did not always show the same results as the IPW analysis. One explanation might be that the higher number of swollen and tender joints in RA patients at diagnosis resulted in more functional limitations and a lower physical HRQoL compared with PsA patients if they are not matched on disease activity. After 1 year, the crude data showed similar results as the IPW analysis in the comparison of RA-tREACH and PsA-DEPAR patients. In contrast to the IPW-weighted analysis, the crude data did not show a difference in the HAQ-DI and SF-36 PCS between RA-EAC and PsA-EAC patients after 1 year. Thus, in daily practice, RA patients seem to have worse physical functioning than oligoarticular and polyarticular PsA patients at diagnosis, but after 1 year, these differences diminish and PROs are similar between RA and PsA patients.

With the currently advocated dual treat-to-target management approach in mind, aiming for control of inflammation and control of disease impact, it is essential that healthcare professionals are aware of the possible (continuous) mental health issues in PsA patients even after treatment.^9 10^ Especially in PsA patients with severe psoriasis and enthesitis, it may be important to be alert for the presence of psychological symptoms during outpatient visits and to refer if necessary.

A strength of our study is that—to our knowledge—this is the first study that has matched large RA and PsA cohorts on multiple patient characteristics, clinical features and PROs, making RA and PsA patients comparable. One previous study also used matching statistics, but only matched for disease duration.^12^ Second, we applied IPW, which ensured that no patients were excluded during the matching process.^32^ We also included several of the ICHOM-recommended PRO domains (pain, fatigue, activity limitations and health impact) to compare the impact of the disease on the lives of patients with inflammatory arthritis. This enabled us to show the difference in disease impact in various domains. Moreover, we used data from two separate regions in the Netherlands. In this study, RA data from a randomised-controlled trial with a fixed medication protocol were compared with PsA data from an observational cohort study without a prespecified medication protocol, which could have influenced our results after 1 year of treatment. RA-tREACH patients may achieve joint remission more quickly, possibly resulting in a lower health impact. A randomised-controlled trial also includes a more selected patient population than a cohort study. To overcome this issue, we validated our results in an independent cohort with both RA and PsA patients, who were treated according to (inter)national guidelines, from another region in the Netherlands. The results from both regions showed some differences both at diagnosis and after 1 year. As discussed previously, we cannot be sure what caused these differences, but it might be due to regional differences in treatment protocols, the relatively small number of PsA patients in the EAC, or differences in (unmeasured) covariates that may influence outcomes. For example, we have no data on the presence of enthesitis or the severity of psoriasis in the EAC. Moreover, comorbidities are known to influence HRQoL scores.^46^ Unfortunately, we do not have data available on (differences in) comorbidities in the different cohorts.

Our study also has some limitations. To satisfy the balancing property, we only included PsA patients with oligoarthritis or polyarthritis, meaning that patients with enthesitis, monarthritis, dactylitis or axial disease as main clinical symptoms were excluded from our analysis. Previous research has shown that PsA patients with enthesitis and axial disease are not necessarily comparable to those with arthritis with respect to PROs.^8 41^ For example, PsA patients with the enthesitis phenotype score higher on fatigue and depression and anxiety than other PsA subtypes.^8^ Therefore, our results only apply to PsA patients with an oligoarthritis or polyarthritis phenotype and cannot be generalised to the whole PsA patient population. Although we tried to match the RA and PsA patients as closely as possible, the requirements for the matching statistics were not always met, especially in the EAC. For example, some variables exceeded the standardised bias of >10% after propensity score matching. To overcome this problem, a double robust IPW was performed in which all covariates with a bias >10% were included.^35^ In addition, analyses were repeated with trimmed propensity scores to improve covariate balance. As mentioned before, there may still be unmeasured confounding, but we managed to get Rubin’s R within the recommended range of 0.5–2 and had a balanced set for our final analyses.^47^ In the trimmed analysis of the EAC, a large number of RA patients were trimmed because of extreme propensity scores. Trimming reduces the variance of the estimates and is thought to reduce bias due to (unmeasured) confounding.^39^ However, reducing the number of patients by trimming may result in a selective group of patients, thereby limiting the generalisability of the results. Finally, we did not take differences in treatment into account in our analyses. In the tREACH and DEPAR, the proportion of patients using a b/tsDMARD was similar during the first year after diagnosis. Surprisingly, patients treated with b/tsDMARDs had worse PROs, except for the SF-36 MCS, than those who did not receive b/tsDMARD treatment. This likely reflects a more severe disease in the patients who required b/tsDMARD treatment. Additional adjustment for b/tsDMARD use did not change the results of the double robust IPW. No data were available in the EAC on differences in treatment between RA and PsA patients.

In conclusion, the disease impact of RA patients is similar to that of matched PsA patients in most domains. However, at baseline, PsA-DEPAR patients had worse mental HRQoL compared with RA-tREACH patients. After 1 year of treatment, PsA-DEPAR patients still had a slightly worse mental HRQoL. This could possibly be due to the additional disease manifestations in PsA, including enthesitis and psoriasis. Aforementioned was not found in the EAC, but PsA-EAC patients did experience more functional limitations after 1 year than RA-EAC patients. Further research in a larger PsA cohort is needed to understand the causes of these differences. Nevertheless, healthcare providers should be aware of the potential differences in disease impact between RA and PsA patients, especially with respect to mental health and functioning.

## Supplementary material

10.1136/rmdopen-2024-005143 online supplemental file 1

## Data Availability

Data are available on reasonable request.
