# Supplementary material for: Rheumatoid arthritis and psoriatic arthritis: is the disease impact different? A large matching study at diagnosis and after 1 year of treatment
Source: RMD Open. 2025 Mar 12;11(1):e005143. doi: 10.1136/rmdopen-2024-005143 (PMC13059954; doi:10.1136/rmdopen-2024-005143)
Supplement: online supplemental file 1 [file rmdopen-11-1-s001.docx]

**Supplementary materials**

**Supplementary table S1.** Missing data per PRO at diagnosis and after 1 year

|  | Number of patients | Pain | Fatigue | HAQ-DI | General health | SF-36 PCS | SF-36 MCS |
| --- | --- | --- | --- | --- | --- | --- | --- |
|  | **Baseline** | | | | | | |
| tREACH (RA) | 391 | 6 (2) | 3 (1) | 5 (1) | 1 (0) | 14 (4) | 14 (4) |
| DEPAR (PsA) | 416 | 31 (7) | 16 (4) | 7 (2) | 13 (3) | 9 (2) | 9 (2) |
| EAC (RA) | 702 | 11 (2) | 14 (2) | 26 (4) | 11 (2) | 92 (13) | 92 (13) |
| EAC (PsA) | 99 | 0 (0) | 0 (0) | 4 (4) | 0 (0) | 10 (10) | 10 (10) |
|  | **1 year** | | | | | | |
| tREACH (RA) | 368 | 80 (22) | 72 (20) | 73 (20) | 44 (12) | 91 (25) | 91 (25) |
| DEPAR (PsA) | 302 | 23 (8) | 20 (7) | 19 (6) | 13 (4) | 17 (6) | 17 (6) |
| EAC (RA) | 463 | 8 (2) | 11 (2) | 57 (12) | 8 (2) | 88 (19) | 88 (19) |
| EAC (PsA) | 68 | 0 (0) | 0 (0) | 14 (21) | 1 (1) | 15 (22) | 15 (22) |

Results shown are the n(%) of missing data per PRO in each of the included cohorts.

*Abbreviations:* *DEPAR, Dutch southwest Early PsA cohort; EAC: Leiden Early Arthritis Clinic; HAQ-DI, Health Assessment Questionnaire – Disability Index; PRO, patient-reported outcome; PsA, psoriatic arthritis; RA, rheumatoid arthritis; SF36-MCS, 36-item Short Form Health Survey Mental Component Score; SF-36 PCS, 36-item Short Form Health Survey Physical Component Score; tREACH: treatment in the Rotterdam Early Arthritis CoHort trial.*

**Supplementary table S2.** Overview of how SJC, TJC and CRP were used to estimate the propensity score at baseline, at 1 year, and after trimming

|  | **SJC** | **TJC** | **CRP** |
| --- | --- | --- | --- |
| **Baseline** |  |  |  |
| tREACH vs DEPAR | SJC continuous | TJC categorized  ≤1, 2-4, ≥5 | CRP dichotomized  ≤10 and >10 |
| EAC vs EAC | SJC categorized  ≤1, 2-4, ≥5 | TJC categorized  ≤1, 2-4, ≥5 | CRP dichotomized  ≤10 and >10 |
| **1 year** |  |  |  |
| tREACH vs DEPAR | SJC categorized  ≤1, 2-4, ≥5 | TJC categorized  ≤1, 2-4, ≥5  + TJC exponentiated to the power of 3 | CRP dichotomized  ≤10 and >10 |
| EAC vs EAC | SJC categorized  ≤1, 2-4, ≥5 | TJC categorized  ≤1, 2-4, ≥5  + TJC exponentiated to the power of 2 | CRP dichotomized  ≤10 and >10 |
| **Baseline, trimmed PS** |  |  |  |
| tREACH vs DEPAR | SJC continuous | TJC categorized  ≤1, 2-4, ≥5 | CRP continuous  + CRP dichotomized ≤10 and >10  + CRP exponentiated to the power of 2 |
| EAC vs EAC | SJC continuous | TJC categorized  ≤1, 2-4, ≥5 | CRP continuous |
| **1 year, trimmed PS** |  |  |  |
| tREACH vs DEPAR | SJC continuous | TJC categorized  ≤1, 2-4, ≥5 | CRP dichotomized  ≤10 and >10 |
| EAC vs EAC | SJC continuous  + SJC categorized  ≤1, 2-4, ≥5 | TJC categorized  ≤1, 2-4, ≥5 | CRP continuous  + CRP dichotomized  ≤10 and >10 |

In order to achieve better matching between RA and PsA, SJC and TJC were categorized into ≤1, 2-4 or ≥5 tender joints, and CRP was dichotomized into ≤10 and >10. In addition, in some propensity scores, TJC was exponentiated to the power of 2 or 3, and CRP was exponentiated to the power of 2. For RA the swollen joint count with 44 joints and tender joint count with 53 joints were used, whereas for PsA the swollen joint count with 66 joints and tender joint count with 68 joints were used.

In the trimmed analyses, propensity scores were discarded if they were outside the range [0.1 - 0.9] to decrease the variance of the estimates and to reduce bias from extreme propensity scores. After trimming, the propensity scores were re-estimated to improve covariate balance.

*Abbreviations: CRP, C-reactive protein; DEPAR, Dutch southwest Early Psoriatic Arthritis cohort; EAC, Leiden Early Arthritis Clinic; PS, propensity score; SJC, swollen joint count; TJC, tender joint count; and tREACH, treatment in the Rotterdam Early Arthritis CoHort trial.*

**Supplementary table S3.** Baseline characteristics before and after IPW for RA (tREACH) and PsA (DEPAR) patients after 1 year of treatment

|  | **Before IPW** | | | **After IPW** | |
| --- | --- | --- | --- | --- | --- |
|  | **RA**  **(n=368)** | **PsA**  **(n=377)** | **SB**  **(%)** | **PsA**  **(n=377)** | **SB**  **(%)** |
| **Demographic characteristics** |  |  |  |  |  |
| Age (years) | 53.4 | 51.3 | 14.7 | 53.8 | -2.8 |
| Sex, female (%) | 67.3 | 47.5 | 40.8 | 65.4 | 4.0 |
| Symptom duration (months) | 5.3 | 31.2 | -61.3 | 5.4 | -0.1 |
| Current smoker (%) | 29.8 | 22.1 | 17.5 | 30.5 | -1.7 |
| Education level* (%) |  |  |  |  |  |
| Low | 56.9 | 40.0 | 34.8 | 56.5 | 0.9 |
| Intermediate | 27.5 | 33.3 | -12.6 | 26.0 | 3.2 |
| High | 15.6 | 27.0 | -28.0 | 17.5 | -4.7 |
| **Disease activity** |  |  |  |  |  |
| SJC-44/66 |  |  |  |  |  |
| SJC-44/66 ≤1 (%) | 5.9 | 20.7 | -44.4 | 6.6 | -1.9 |
| SJC-44/66 2-4 (%) | 24.7 | 39.2 | -31.5 | 21.0 | 8.0 |
| SJC-44/66 ≥5 (%) | 69.4 | 40.1 | 61.4 | 72.4 | -6.5 |
| TJC-53/68 |  |  |  |  |  |
| TJC-53/68 ≤1 (%) | 7.8 | 18.0 | -30.6 | 7.2 | 1.9 |
| TJC-53/68 2-4 (%) | 15.9 | 31.3 | -36.9 | 15.0 | 2.0 |
| TJC-53/68 ≥5 (%) | 76.3 | 50.7 | 55.1 | 77.8 | -3.2 |
| CRP |  |  |  |  |  |
| CRP ≤10 | 59.1 | 66.9 | -16.2 | 64.4 | **-10.9** |
| CRP >10 | 40.9 | 33.1 | 16.2 | 35.6 | **10.9** |
| **PROs at baseline** |  |  |  |  |  |
| VAS pain | 54.1 | 50.1 | 14.8 | 54.5 | -1.6 |
| VAS fatigue | 50.2 | 49.2 | 3.9 | 50.1 | 0.6 |
| HAQ-DI | 1.01 | 0.81 | 32.8 | 0.99 | 3.4 |
| VAS general health | 50.2 | 48.9 | 5.5 | 49.8 | 1.6 |
| SF-36 PCS | 35.3 | 38.6 | -36.5 | 35.7 | -4.5 |
| SF-36 MCS | 50.6 | 47.7 | 28.8 | 50.4 | 3.9 |
| **Matching statistics** | **Before IPW** | | | **After IPW** | |
| Mean bias |  | 30.3 |  | 4.3 | |
| Rubin’s B |  | 79.3 |  | 24.2 | |
| Rubin’s R |  | 0.03 |  | 1.71 | |

Results shown are means or as stated otherwise

*Education level was determined with the scale of the Organisation for Economic Co-operation and Development: low, below upper secondary level; intermediate, upper secondary level; high, tertiary education.

In order to achieve better matching between RA and PsA, SJC and TJC were categorized into ≤1, 2-4 or ≥5 swollen/tender joints, and CRP was dichotomized into ≤10 and >10. For RA the SJC44 and TJC53 were used, whereas for PsA the SJC66 and TJC68 were used.

SB is the absolute standardized bias, also called absolute standardized mean difference (in %). Rubin’s B is the absolute standardized difference of means between the RA and PsA group. Rubin’s R is the ratio of the group variances of the propensity score of the two groups. When optimal balance is achieved SB is below 10%, Rubin’s B is below 25, and Rubin’s R is between 0.5 and 2. Since optimal balance was not achieved, in the double robust method, an extra correction was done on top of the weighting for the variable with a SB above 10% after weighting. This variable was CRP and is indicated in bold.

*Abbreviations: CRP, C-reactive protein; DEPAR, Dutch southwest Early PsA cohort; HAQ-DI, Health Assessment Questionnaire – Disability Index; IPW, inverse probability weighting; PsA, psoriatic arthritis; RA, rheumatoid arthritis; SB, standardized bias; SF36-MCS, 36-item Short Form Health Survey Mental Component Score; SF-36 PCS, 36-item Short Form Health Survey Physical Component Score; SJC-44, 44 swollen joint count; SJC-66, 66 swollen joint count; TJC-53, 53 tender joint count; TJC-68, 68 tender joint count; and* *tREACH, treatment in the Rotterdam Early Arthritis CoHort trial.*

**Supplementary table S4.** Baseline characteristics before and after IPW for RA (EAC) and PsA (EAC) patients after 1 year of treatment

|  | **Before IPW** | | | **After IPW** | |
| --- | --- | --- | --- | --- | --- |
|  | **RA**  **(n=593)** | **PsA**  **(n=80)** | **SB**  **(%)** | **PsA**  **(n=80)** | **SB**  **(%)** |
| **Demographic characteristics** |  |  |  |  |  |
| Age (years) | 58.9 | 49.4 | 64.5 | 60.4 | **-10.2** |
| Sex, female (%) | 63.4 | 40.1 | 47.7 | 72.3 | **-18.4** |
| Symptom duration (months) | 7.0 | 9.8 | -16.1 | 5.7 | 7.8 |
| Current smoker (%) | 21.4 | 18.5 | 7.1 | 28.3 | **-17.3** |
| **Disease activity** |  |  |  |  |  |
| SJC-44/66 |  |  |  |  |  |
| SJC-44/66 ≤1 (%) | 14.4 | 25.4 | -27.6 | 10.6 | 9.7 |
| SJC-44/66 2-4 (%) | 29.9 | 45.4 | -32.2 | 37.9 | **-16.6** |
| SJC-44/66 ≥5 (%) | 55.6 | 29.2 | 55.3 | 51.5 | 8.6 |
| TJC-53/68 |  |  |  |  |  |
| TJC-53/68 ≤1 (%) | 8.2 | 15.4 | -22.3 | 6.5 | 5.5 |
| TJC-53/68 2-4 (%) | 21.0 | 29.2 | -18.9 | 21.6 | -1.2 |
| TJC-53/68 ≥5 (%) | 70.7 | 55.4 | 32.1 | 72.0 | -2.6 |
| CRP |  |  |  |  |  |
| CRP ≤10 | 53.4 | 64.9 | -23.6 | 55.2 | -3.6 |
| CRP >10 | 46.6 | 35.1 | 23.6 | 44.8 | 3.6 |
| **PROs at baseline** |  |  |  |  |  |
| VAS pain | 57.9 | 57.8 | 0.3 | 52.5 | **22.7** |
| VAS fatigue | 49.2 | 42.0 | 23.5 | 46.1 | **10.3** |
| HAQ-DI | 1.03 | 0.67 | 57.0 | 0.97 | 8.9 |
| VAS general health | 42.9 | 33.4 | 39.5 | 38.5 | **18.1** |
| SF-36 PCS | 35.4 | 41.0 | -61.3 | 35.0 | 4.1 |
| SF-36 MCS | 47.4 | 50.3 | -27.7 | 48.3 | -8.7 |
| **Matching statistics** | **Before IPW** | | | **After IPW** | |
| Mean bias |  | 32.6 |  | 10.5 | |
| Rubin’s B |  | 118.3 |  | 52.2 | |
| Rubin’s R |  | 1.12 |  | 1.47 | |

Results shown are means or as stated otherwise

In order to achieve better matching between RA and PsA, SJC and TJC were categorized into ≤1, 2-4 or ≥5 swollen/tender joints, and CRP was dichotomized into ≤10 and >10. For RA the swollen joint count with 44 joints and tender joint count with 53 joints were used, whereas for PsA the swollen joint count with 66 joints and tender joint count with 68 joints were used.

SB is the absolute standardized bias, also called absolute standardized mean difference (in %). Rubin’s B is the absolute standardized difference of means between the RA and PsA group. Rubin’s R is the ratio of the group variances of the propensity score of the two groups. When optimal balance is achieved SB is below 10%, Rubin’s B is below 25, and Rubin’s R is between 0.5 and 2. Since optimal balance was not achieved, in the double robust method, an extra correction was done on top of the weighting for the variables with a SB above 10% after weighting. These variables were age, sex, smoking status, swollen joint count, baseline pain, fatigue and general health and are indicated in bold.

*Abbreviations:* *CRP, C-reactive protein; EAC: Leiden Early Arthritis Clinic; HAQ-DI, Health Assessment Questionnaire – Disability Index; IPW, inverse probability weighting; PsA, psoriatic arthritis; RA, rheumatoid arthritis; SB, standardized bias; SF36-MCS, 36-item Short Form Health Survey Mental Component Score; SF-36 PCS, 36-item Short Form Health Survey Physical Component Score; SJC-44, 44 swollen joint count; SJC-66, 66 swollen joint count; TJC-53, 53 tender joint count; and TJC-68, 68 tender joint count.*

**Supplementary table S5.** Crude estimates of PRO-scores for RA-tREACH and PsA-DEPAR patients after 1 year of treatment, stratified for the use of b/tsDMARDs during the first year after diagnosis

| **PROs** | **RA – tREACH** | | | | **PsA – DEPAR** | | | |
| --- | --- | --- | --- | --- | --- | --- | --- | --- |
|  | **b/tsDMARD (n=117)** | | **no b/tsDMARD (n=251)** | | **b/tsDMARD (n=99)** | | **no b/tsDMARD (n=203)** | |
| **VAS pain** | 36.8 | (27.2) | 20.7 | (24.3) | 37.5 | (29.6) | 28.8 | (26.5) |
| **VAS fatigue** | 53.2 | (25.9) | 38.3 | (27.5) | 47.9 | (28.1) | 40.5 | (28.7) |
| **HAQ-DI** | 0.87 | (0.57) | 0.47 | (0.55) | 0.64 | (0.63) | 0.57 | (0.56) |
| **VAS general health** | 34.1 | (22.5) | 24.4 | (19.6) | 34.9 | (27.6) | 27.0 | (23.9) |
| **SF-36 PCS** | 38.1 | (10.3) | 45.2 | (9.7) | 42.1 | (10.4) | 44.4 | (8.9) |
| **SF-36 MCS** | 52.8 | (9.8) | 52.9 | (8.9) | 47.5 | (10.9) | 49.2 | (10.4) |

Results shown are the mean (sd).

*Abbreviations: b/tsDMARDs, biological or targeted synthetic disease-modifying anti-rheumatic drugs; DEPAR, Dutch southwest Early PsA cohort; HAQ-DI, Health Assessment Questionnaire – Disability Index; PRO, patient-reported outcome; PsA, psoriatic arthritis; RA, rheumatoid arthritis; SF36-MCS, 36-item Short Form Health Survey Mental Component Score; SF-36 PCS, 36-item Short Form Health Survey Physical Component Score; tREACH: treatment in the Rotterdam Early Arthritis CoHort trial; and VAS, Visual Analogue Scale.*

**Supplementary table S6.** Baseline characteristics before and after IPW for RA (tREACH) and PsA (DEPAR) patients at diagnosis, after trimming extreme propensity scores

|  | **Before IPW** | | | **After IPW** | |
| --- | --- | --- | --- | --- | --- |
|  | **RA**  **(n=352)** | **PsA**  **(n=282)** | **SB**  **(%)** | **PsA**  **(n=282)** | **SB**  **(%)** |
| **Demographic characteristics** |  |  |  |  |  |
| Age (years) | 52.7 | 51.5 | 8.9 | 53.0 | -2.2 |
| Sex, female (%) | 66.3 | 47.7 | 38.3 | 63.9 | 4.9 |
| Symptom duration (months) | 5.5 | 15.3 | -38.6 | 4.9 | 2.5 |
| Current smoker (%) | 29.6 | 24.0 | 12.8 | 29.2 | 0.9 |
| Education level* (%) |  |  |  |  |  |
| Low | 54.7 | 41.5 | 26.7 | 53.3 | 2.9 |
| Intermediate | 28.8 | 31.8 | -6.5 | 30.7 | -4.2 |
| High | 16.5 | 26.7 | -25.1 | 16.0 | 1.2 |
| **Disease activity** |  |  |  |  |  |
| SJC-44/66 | 7.5 | 4.9 | 56.6 | 7.2 | 7.6 |
| TJC-53/68 |  |  |  |  |  |
| TJC-53/68 ≤1 (%) | 8.6 | 17.3 | -26.0 | 6.3 | 7.0 |
| TJC-53/68 2-4 (%) | 17.2 | 32.0 | -34.8 | 18.1 | -2.0 |
| TJC-53/68 ≥5 (%) | 74.2 | 50.7 | 49.8 | 75.6 | -3.1 |
| CRP | 16.1 | 11.5 | 20.8 | 16.3 | -0.5 |
| **Matching statistics** | **Before IPW** | | | **After IPW** | |
| Mean bias |  | 25.0 |  | 3.1 | |
| Rubin’s B |  | 106.3 |  | 20.9 | |
| Rubin’s R |  | 0.74 |  | 0.67 | |

Results shown are means or as stated otherwise.

*Education level was defined according to the Organisation for Economic Co-operation and Development: low, below upper secondary level; intermediate, upper secondary level; high, tertiary education.

In order to achieve better matching between RA and PsA, TJC was categorized into ≤1, 2-4 or ≥5 tender joints. For RA the swollen joint count with 44 joints and tender joint count with 53 joints were used, whereas for PsA the swollen joint count with 66 joints and tender joint count with 68 joints were used.

SB is the absolute standardized bias, also called absolute standardized mean difference (in %). Rubin’s B is the absolute standardized difference of means between the RA and PsA group. Rubin’s R is the ratio of the group variances of the propensity score of the two groups. When optimal balance is achieved SB is below 10%, Rubin’s B is below 25, and Rubin’s R is between 0.5 and 2. Since optimal balance was achieved after trimming propensity scores outside the range [0.1 - 0.9] and re-estimating the propensity score, no extra correction was needed after weighting.

*Abbreviations: CRP, C-reactive protein; DEPAR, Dutch southwest Early PsA cohort; IPW, inverse probability weighting; PsA, psoriatic arthritis; RA, rheumatoid arthritis; SB, standardized bias; SJC-44, 44 swollen joint count; SJC-66, 66 swollen joint count; TJC-53, 53 tender joint count; TJC-68, 68 tender joint count; and tREACH, treatment in the Rotterdam Early Arthritis CoHort trial.*

**Supplementary table S7.** Baseline characteristics before and after IPW for RA (EAC) and PsA (EAC) patients at diagnosis, after trimming extreme propensity scores

|  | **Before IPW** | | | **After IPW** | |
| --- | --- | --- | --- | --- | --- |
|  | **RA**  **(n=277)** | **PsA**  **(n=76)** | **SB**  **(%)** | **PsA**  **(n=76)** | **SB**  **(%)** |
| **Demographic characteristics** |  |  |  |  |  |
| Age (years) | 53.3 | 46.3 | 48.8 | 51.6 | **12.2** |
| Sex, female (%) | 47.2 | 30.7 | 34.3 | 45.3 | 4.0 |
| Symptom duration (months) | 6.7 | 11.0 | -26.0 | 6.0 | 4.5 |
| Current smoker (%) | 18.3 | 14.9 | 9.3 | 15.3 | 8.1 |
| **Disease activity** |  |  |  |  |  |
| SJC-44/66 | 5.2 | 3.7 | 34.6 | 3.5 | **40.0** |
| TJC-53/68 |  |  |  |  |  |
| TJC-53/68 ≤1 (%) | 11.4 | 17.8 | -17.9 | 20.2 | **-24.9** |
| TJC-53/68 2-4 (%) | 25.6 | 30.8 | -11.6 | 25.7 | -0.2 |
| TJC-53/68 ≥5 (%) | 62.9 | 51.4 | 23.4 | 54.1 | **18.0** |
| CRP | 16.1 | 10.7 | 27.4 | 14.8 | 6.9 |
| **Matching statistics** | **Before IPW** | | | **After IPW** | |
| Mean bias |  | 25.9 |  | 13.2 | |
| Rubin’s B |  | 89.3 |  | 35.0 | |
| Rubin’s R |  | 0.73 |  | 1.67 | |

Results shown are means or as stated otherwise.

In order to achieve better matching between RA and PsA, TJC was categorized into ≤1, 2-4 or ≥5 tender joints. For RA the swollen joint count with 44 joints and tender joint count with 53 joints were used, whereas for PsA the swollen joint count with 66 joints and tender joint count with 68 joints were used.

SB is the absolute standardized bias, also called absolute standardized mean difference (in %). Rubin’s B is the absolute standardized difference of means between the RA and PsA group. Rubin’s R is the ratio of the group variances of the propensity score of the two groups. When optimal balance is achieved SB is below 10%, Rubin’s B is below 25, and Rubin’s R is between 0.5 and 2. Since optimal balance was not achieved after trimming propensity scores outside the range [0.1 - 0.9] and re-estimating the propensity score, in the double robust method, an extra correction was done on top of the weighting for the variables with a SB above 10% after weighting. These variables were age, swollen joint count and tender joint count and are indicated in bold.

*Abbreviations: CRP, C-reactive protein; EAC, Leiden Early Arthritis Clinic; IPW, inverse probability weighting; PsA, psoriatic arthritis; RA, rheumatoid arthritis; SB, standardized bias; SJC-44, 44 swollen joint count; SJC-66, 66 swollen joint count; TJC-53, 53 tender joint count; and TJC-68, 68 tender joint count.*

**Supplementary figure S1.** Differences in patient-reported outcomes between RA and PsA patients at diagnosis, after trimming^1^ extreme propensity scores

Values shown are the mean differences in PROs between RA and PsA at diagnosis with the corresponding 95% confidence interval. For readability purposes negative differences in SF-36 PCS and MCS values are shown as positive differences and vice versa, so that a negative difference is in favour of RA and a positive difference in favour of PsA. Since optimal balance was not achieved in the RA-EAC vs PsA-EAC, in the double robust method, an extra correction was done on top of the weighting for age, and baseline swollen joint count and tender joint count. In the comparison of the RA-tREACH vs PsA-DEPAR optimal balance was achieved and no additional corrections were needed.

^1^In the analysis with the tREACH 173 patients were excluded (39 RA patients and 134 PsA patients), while in the analysis with the EAC 448 patients were excluded (425 RA patients and 23 PsA patients).

*Abbreviations: DEPAR, Dutch southwest Early PsA cohort; EAC, Leiden Early Arthritis Clinic; HAQ-DI, Health Assessment Questionnaire – Disability Index; IPW, inverse probability weighting; PsA, psoriatic arthritis; RA, rheumatoid arthritis; SF36-MCS, 36-item Short Form Health Survey Mental Component Score; SF-36 PCS, 36-item Short Form Health Survey Physical Component Score; tREACH: treatment in the Rotterdam Early Arthritis CoHort trial; and VAS, Visual Analogue Scale.*

**Supplementary table S8.** Baseline characteristics before and after IPW for RA (tREACH) and PsA (DEPAR) patients after 1 year of treatment, after trimming extreme propensity scores

|  | **Before IPW** | | | **After IPW** | |
| --- | --- | --- | --- | --- | --- |
|  | **RA**  **(n=311)** | **PsA**  **(n=249)** | **SB**  **(%)** | **PsA**  **(n=249)** | **SB**  **(%)** |
| **Demographic characteristics** |  |  |  |  |  |
| Age (years) | 53.3 | 51.7 | 11.7 | 53.2 | 1.0 |
| Sex, female (%) | 63.3 | 48.0 | 31.2 | 63.0 | 0.6 |
| Symptom duration (months) | 5.5 | 16.9 | -41.1 | 5.3 | 0.6 |
| Current smoker (%) | 29.2 | 22.7 | 14.7 | 28.4 | 1.7 |
| Education level* (%) |  |  |  |  |  |
| Low | 54.1 | 41.4 | 25.7 | 53.3 | 1.7 |
| Intermediate | 28.8 | 31.8 | -6.4 | 28.6 | 0.5 |
| High | 17.1 | 26.8 | -23.8 | 18.1 | -2.6 |
| **Disease activity** |  |  |  |  |  |
| SJC-44/66 | 8.2 | 5.0 | 59.1 | 7.8 | 7.7 |
| TJC-53/68 |  |  |  |  |  |
| TJC-53/68 ≤1 (%) | 8.8 | 17.1 | -24.9 | 6.6 | 6.4 |
| TJC-53/68 2-4 (%) | 18.1 | 31.8 | -32.1 | 17.4 | 1.6 |
| TJC-53/68 ≥5 (%) | 73.2 | 51.2 | 46.5 | 76.0 | -6.0 |
| CRP |  |  |  |  |  |
| CRP ≤10 | 59.6 | 66.6 | -14.5 | 61.2 | -3.3 |
| CRP >10 | 40.4 | 33.4 | 14.5 | 38.8 | 3.3 |
| **PROs at baseline** |  |  |  |  |  |
| VAS pain | 52.9 | 50.1 | 10.1 | 54.6 | -6.3 |
| VAS fatigue | 48.4 | 47.4 | 3.8 | 48.0 | 1.3 |
| HAQ-DI | 0.96 | 0.80 | 25.5 | 0.93 | 3.9 |
| VAS general health | 49.6 | 48.1 | 6.1 | 48.7 | 3.7 |
| SF-36 PCS | 36.4 | 38.9 | -28.3 | 36.8 | -4.6 |
| SF-36 MCS | 49.9 | 48.1 | 17.4 | 49.5 | 3.9 |
| **Matching statistics** | **Before IPW** | | | **After IPW** | |
| Mean bias |  | 23.0 |  | 3.2 | |
| Rubin’s B |  | 102.7 |  | 16.2 | |
| Rubin’s R |  | 0.92 |  | 1.53 | |

Results shown are means or as stated otherwise.

*Education level was defined according to the Organisation for Economic Co-operation and Development: low, below upper secondary level; intermediate, upper secondary level; high, tertiary education.

In order to achieve better matching between RA and PsA, TJC was categorized into ≤1, 2-4 or ≥5 tender joints, and CRP was dichotomized into ≤10 and >10. For RA the swollen joint count with 44 joints and tender joint count with 53 joints were used, whereas for PsA the swollen joint count with 66 joints and tender joint count with 68 joints were used.

SB is the absolute standardized bias, also called absolute standardized mean difference (in %). Rubin’s B is the absolute standardized difference of means between the RA and PsA group. Rubin’s R is the ratio of the group variances of the propensity score of the two groups. When optimal balance is achieved SB is below 10%, Rubin’s B is below 25, and Rubin’s R is between 0.5 and 2. Since optimal balance was achieved after trimming propensity scores outside the range [0.1 - 0.9] and re-estimating the propensity score, no extra correction was needed after weighting.

*Abbreviations: CRP, C-reactive protein; DEPAR, Dutch southwest Early PsA cohort; HAQ-DI, Health Assessment Questionnaire – Disability Index; IPW, inverse probability weighting; PsA, psoriatic arthritis; RA, rheumatoid arthritis; SB, standardized bias; SF36-MCS, 36-item Short Form Health Survey Mental Component Score; SF-36 PCS, 36-item Short Form Health Survey Physical Component Score; SJC-44, 44 swollen joint count; SJC-66, 66 swollen joint count; TJC-53, 53 tender joint count; TJC-68, 68 tender joint count; tREACH, treatment in the Rotterdam Early Arthritis CoHort trial; and VAS, Visual Analogue Scale.*

**Supplementary table S9.** Baseline characteristics before and after IPW for RA (EAC) and PsA (EAC) patients after 1 year of treatment, after trimming extreme propensity scores

|  | **Before IPW** | | | **After IPW** | |
| --- | --- | --- | --- | --- | --- |
|  | **RA**  **(n=215)** | **PsA**  **(n=67)** | **SB**  **(%)** | **PsA**  **(n=67)** | **SB**  **(%)** |
| **Demographic characteristics** |  |  |  |  |  |
| Age (years) | 55.5 | 47.0 | 57.4 | 50.1 | **36.3** |
| Sex, female (%) | 54.7 | 33.6 | 43.4 | 47.1 | **15.6** |
| Symptom duration (months) | 6.7 | 10.9 | -24.7 | 6.7 | 0.2 |
| Current smoker (%) | 20.1 | 16.5 | 9.4 | 23.6 | -8.8 |
| **Disease activity** |  |  |  |  |  |
| SJC-44/66 | 5.7 | 3.6 | 47.3 | 3.5 | **49.1** |
| TJC-53/68 |  |  |  |  |  |
| TJC-53/68 ≤1 (%) | 10.7 | 16.5 | -17.0 | 10.9 | -0.7 |
| TJC-53/68 2-4 (%) | 25.2 | 31.2 | -13.4 | 36.9 | **-26.1** |
| TJC-53/68 ≥5 (%) | 64.1 | 52.3 | 24.1 | 52.2 | **24.4** |
| CRP | 17.0 | 10.8 | 29.6 | 17.1 | -0.8 |
| **PROs at baseline** |  |  |  |  |  |
| VAS pain | 57.3 | 58.0 | -3.1 | 55.7 | 6.7 |
| VAS fatigue | 44.1 | 40.5 | 11.7 | 33.8 | **33.1** |
| HAQ-DI | 0.81 | 0.59 | 37.0 | 0.73 | **14.2** |
| VAS general health | 37.5 | 31.8 | 24.4 | 34.0 | **14.8** |
| SF-36 PCS | 38.4 | 42.6 | -44.8 | 38.8 | -3.7 |
| SF-36 MCS | 49.0 | 51.3 | -23.6 | 49.8 | -7.6 |
| **Matching statistics** | **Before IPW** | | | **After IPW** | |
| Mean bias |  | 27.0 |  | 17.0 | |
| Rubin’s B |  | 109.2 |  | 49.5 | |
| Rubin’s R |  | 0.67 |  | 2.11 | |

Results shown are means or as stated otherwise.

In order to achieve better matching between RA and PsA, TJC was categorized into ≤1, 2-4 or ≥5 tender joints. For RA the swollen joint count with 44 joints and tender joint count with 53 joints were used, whereas for PsA the swollen joint count with 66 joints and tender joint count with 68 joints were used.

SB is the absolute standardized bias, also called absolute standardized mean difference (in %). Rubin’s B is the absolute standardized difference of means between the RA and PsA group. Rubin’s R is the ratio of the group variances of the propensity score of the two groups. When optimal balance is achieved SB is below 10%, Rubin’s B is below 25, and Rubin’s R is between 0.5 and 2. Since optimal balance was not achieved after trimming propensity scores outside the range [0.1 - 0.9] and re-estimating the propensity score, in the double robust method, an extra correction was done on top of the weighting for the variables with a SB above 10% after weighting. These variables were age, sex, baseline swollen joint count, tender joint count, fatigue, HAQ-DI and general health.

*Abbreviations: CRP, C-reactive protein; EAC, Leiden Early Arthritis Clinic; HAQ-DI, Health Assessment Questionnaire – Disability Index; IPW, inverse probability weighting; PsA, psoriatic arthritis; RA, rheumatoid arthritis; SB, standardized bias; SF36-MCS, 36-item Short Form Health Survey Mental Component Score; SF-36 PCS, 36-item Short Form Health Survey Physical Component Score; SJC-44, 44 swollen joint count; SJC-66, 66 swollen joint count; TJC-53, 53 tender joint count; TJC-68, 68 tender joint count; and VAS, Visual Analogue Scale.*


**Supplementary figure S2.** Differences in patient-reported outcomes between RA and PsA patients after 1 year, after trimming^1^ extreme propensity scores
Values shown are the mean differences in PROs between RA and PsA at diagnosis with the corresponding 95% confidence interval. Since optimal balance was not achieved in the RA-EAC vs PsA-EAC, in the double robust method, an extra correction was done on top of the weighting for age, sex, baseline swollen joint count and tender joint count, and baseline general health, fatigue and HAQ-DI. In the comparison of the RA-tREACH vs PsA-DEPAR optimal balance was achieved and no additional corrections were needed.
^1^In the analysis with the tREACH 185 patients were excluded (57 RA patients and 128 PsA patients), while in the analysis with the EAC 399 patients were excluded (378 RA patients and 21 PsA patients).
*Abbreviations: DEPAR, Dutch southwest Early PsA cohort; EAC: Leiden Early Arthritis Clinic; HAQ-DI, Health Assessment Questionnaire – Disability Index; IPW, inverse probability weighting; PsA, psoriatic arthritis; RA, rheumatoid arthritis; SF36-MCS, 36-item Short Form Health Survey Mental Component Score; SF-36 PCS, 36-item Short Form Health Survey Physical Component Score; tREACH: treatment in the Rotterdam Early Arthritis CoHort trial; and VAS, Visual Analogue Scale.*

**Supplementary table S10.** Crude estimates of PRO-scores for RA and PsA patients at diagnosis

| **PROs** | **RA – tREACH**  **(n=391)** | | **PsA – DEPAR**  **(n=416)** | | **RA – EAC**  **(n=702)** | | **PsA – EAC**  **(n=99)** | |
| --- | --- | --- | --- | --- | --- | --- | --- | --- |
| **VAS pain** | 54.0 | (27.6) | 50.6 | (27.0) | 57.6 | (25.0) | 56.3 | (23.3) |
| **VAS fatigue** | 49.8 | (26.8) | 48.7 | (27.3) | 48.6 | (30.4) | 40.0 | (30.5) |
| **HAQ-DI** | 0.99 | (0.66) | 0.80 | (0.55) | 1.03 | (0.64) | 0.68 | (0.58) |
| **VAS general health** | 49.9 | (22.6) | 50.1 | (25.7) | 42.8 | (25.0) | 31.3 | (21.1) |
| **SF-36 PCS** | 35.5 | (9.6) | 38.7 | (8.7) | 35.6 | (9.0) | 40.8 | (9.3) |
| **SF-36 MCS** | 50.9 | (10.8) | 48.0 | (10.6) | 47.5 | (10.8) | 50.6 | (9.3) |

Results shown are the mean (sd).

*Abbreviations: DEPAR, Dutch southwest Early PsA cohort; EAC: Leiden Early Arthritis Clinic; HAQ-DI, Health Assessment Questionnaire – Disability Index; PROs, patient-reported outcomes; PsA, psoriatic arthritis; RA, rheumatoid arthritis; SF36-MCS, 36-item Short Form Health Survey Mental Component Score; SF-36 PCS, 36-item Short Form Health Survey Physical Component Score; tREACH: treatment in the Rotterdam Early Arthritis CoHort trial; and VAS, Visual Analogue Scale.*

**Supplementary table S11.** Crude estimates of PRO-scores for RA and PsA patients after 1 year of treatment

| **PROs** | **RA – tREACH**  **(n=368)** | | **PsA – DEPAR**  **(n=302)** | | **RA – EAC**  **(n=463)** | | **PsA – EAC**  **(n=68)** | |
| --- | --- | --- | --- | --- | --- | --- | --- | --- |
| **VAS pain** | 26.3 | (26.4) | 31.7 | (27.9) | 28.7 | (27.0) | 32.6 | (27.7) |
| **VAS fatigue** | 43.5 | (27.8) | 42.9 | (28.7) | 41.1 | (30.9) | 33.6 | (32.0) |
| **HAQ-DI** | 0.60 | (0.59) | 0.59 | (0.58) | 0.56 | (0.60) | 0.40 | (0.55) |
| **VAS general health** | 27.7 | (21.1) | 29.6 | (25.4) | 34.6 | (23.1) | 27.4 | (21.6) |
| **SF-36 PCS** | 42.8 | (10.5) | 43.7 | (9.4) | 43.7 | (10.1) | 44.4 | (10.0) |
| **SF-36 MCS** | 52.8 | (9.2) | 48.6 | (10.6) | 48.9 | (9.7) | 50.6 | (9.2) |

Results shown are the mean (sd).

*Abbreviations: DEPAR, Dutch southwest Early PsA cohort;* *EAC: Leiden Early Arthritis Clinic;* *HAQ-DI, Health Assessment Questionnaire – Disability Index;* *PRO, patient-reported outcome;* *PsA, psoriatic arthritis; RA, rheumatoid arthritis;* *SF36-MCS, 36-item Short Form Health Survey Mental Component Score; SF-36 PCS, 36-item Short Form Health Survey Physical Component Score;* *tREACH: treatment in the Rotterdam Early Arthritis CoHort trial; and VAS, Visual Analogue Scale.*

**Supplementary table S12.** Crude estimates of SF-36 MCS scores for PsA-DEPAR patients at diagnosis and after 1 year of treatment, by severity of psoriasis and presence/absence of enthesitis

|  | SF-36 MCS | |
| --- | --- | --- |
|  | **Baseline** | **1 year** |
| Psoriasis |  |  |
| BSA ≤3% | 51.5 (41-57) | 52.6 (43-57) |
| BSA >3% | 48.6 (40-56) | 49.7 (39-57) |
| Enthesitis |  |  |
| LEI =0 | 51.5 (43-57) | 52.6 (43-57) |
| LEI >0 | 46.4 (38-55) | 49.1 (40-57) |

Results shown are the median (IQR).

*Abbreviations: BSA, body surface area;* *DEPAR, Dutch southwest Early PsA cohort; LEI, Leeds Enthesitis Index; PsA, psoriatic arthritis; SF36-MCS, 36-item Short Form Health Survey Mental Component Score.*
